# Isothiocyanate-Based Microemulsions Loaded into Biocompatible Hydrogels as Innovative Biofumigants for Agricultural Soils

**DOI:** 10.3390/molecules29163935

**Published:** 2024-08-21

**Authors:** Michele Baglioni, Ilaria Clemente, Gabriella Tamasi, Flavia Bisozzi, Sara Costantini, Giacomo Fattori, Mariangela Gentile, Claudio Rossi

**Affiliations:** 1 Department of Biotechnology, Chemistry and Pharmacy, University of Siena, Via Aldo Moro 2, 53100 Siena, Italy; ilaria.clemente2@unisi.it (I.C.); gabriella.tamasi@unisi.it (G.T.); flavia.bisozzi@student.unisi.it (F.B.); sara.costantini@student.unisi.it (S.C.); giacomo.fattori@student.unisi.it (G.F.); claudio.rossi@unisi.it (C.R.); 2 Centre for Colloid and Surface Science (CSGI), University of Florence, Via della Lastruccia 3, 50019 Sesto Fiorentino, Italy; 3 Department of Life Sciences, University of Siena, Via Aldo Moro 2, 53100 Siena, Italy; mariangela.gentile@unisi.it

**Keywords:** alginate, carboxymethyl cellulose, controlled-release, Tween 80, loading efficiency, release rate, Weibull function

## Abstract

Biofumigation was proposed as an alternative to synthetic pesticides for the disinfection of agricultural soils, in view of the biocidal effect of isothiocyanates (ITCs) released by some vegetal species, like *Brassicaceae*. However, biofumigation also presents limitations; thus, a novel and viable alternative could be the direct introduction of ITCs into agricultural soils as components loaded into biodegradable hydrogels. Thus, in this work, ITCs-based microemulsions were developed, which can be loaded into porous polymer-based hydrogel beads based on sodium alginate (ALG) or sodium carboxymethyl cellulose (CMC). Three ITCs (ethyl, phenyl, and allyl isothiocyanate) and three different surfactants (sodium dodecylsulfate, Brij 35, and Tween 80) were considered. The optimal system was characterized with attenuated ATR-FTIR spectroscopy and differential scanning calorimetry to study how the microemulsion/gels interaction affects the gel properties, such as the equilibrium water content or free water index. Finally, loading and release profiles were studied by means of UV–Vis spectrophotometry. It was found that CMC hydrogel beads showed a slightly more efficient profile of micelles’ release in water with respect to ALG beads. For this reason, and due to the enhanced contribution of Fe(III) to their biocidal properties, CMC-based hydrogels are the most promising in view of the application on real agricultural soils.

## 1. Introduction

Agricultural practices play a major role in ensuring global food availability and the need to boost crop productivity often involves the use of a number of chemicals, among which synthetic pesticides represent a serious concern, due to their detrimental impact on both human health and the environment [1,2,3,4,5,6]. These chemicals, designed to combat pests and diseases, pose risks beyond their intended targets, contaminating soil, water, and food supplies.

Pesticides such as Metam sodium (commercially also known as Vapam); Metam potassium (commercially also known as Tamifum), i.e., dithiocarbamate sodium or potassium salts, respectively; or Dazomet—chemically a 3,5-dimethyl-1,3,5-thiadiazinane-2-thione—have been largely used as soil fumigants against insects, fungi, bacteria, and nematodes. All these chemicals were proposed as milder and more environmentally tolerable substitutes for methyl bromide (bromomethane), a very effective fumigant, which was banned by the Montreal Protocol (1987) [7], due to its high toxicity and, mostly, due to its contribution to stratospheric ozone depletion. The chemical activity of Metam sodium, Metam potassium, and Dazomet is very similar, as they all release methyl isothiocyanate in treated soils. Even if these pesticides represent an improvement over bromomethane, their environmental impact and health concerns are still too high [8,9,10], such that the use of Metam sodium was limited in the EU starting from 2009 [11]. However, in some countries (such as Italy), it was still used in derogation to the regulations, theoretically, only for “essential needs” until 2014, on some crops, such as rice, lettuce, tomatoes, peppers, aubergines, cucurbits, carrots, tuber and stem vegetables, potatoes, tobacco, etc. (DM 11 January 2010, in application of EU Council decision 2009/562/CE)

An interesting alternative strategy to the use of these fumigants was the introduction of biofumigation [12,13,14,15,16,17], a method where fresh plants (mostly *Brassicaceae*) are cultivated, mown, and incorporated into agricultural soils, where they produce, in certain conditions, the same isothiocyanates (ITCs) that are responsible for the biocide activity of the original pesticides. The prominent role of *Brassicaceae* species in this context is due to their high content in glucosinolates, a class of secondary metabolites that can be hydrolyzed to ITCs by the enzyme myrosinase.

In plants, glucosinolates are located in different cellular compartments from myrosinase, but the enzymatic process can be induced by any mechanical tissue damage (grinding, mowing, etc.) [18]. Once initiated, myrosinase-catalyzed hydrolysis of glucosinolates initially involves the cleavage of the thioglucoside bond, resulting in the formation of D-glucose and thiohydroximate-O-sulfonate. The latter is unstable and thus spontaneously rearranges, leading to a wide range of products, such as thiocyanates, nitriles, and, as said, ITCs. These resulting chemicals are involved in defensive mechanisms against soil-borne phytopathogens, fungi, and insects, and their formation is influenced by the reaction conditions, such as pH: ITCs are produced at a neutral pH, while nitriles are produced at an acidic pH [19,20].

In fact, biofumigation exploits the defensive strategy of *Brassicaceae* for agricultural soils’ treatment, in view of the biocidal effect of mostly ITCs. Several studies [20,21,22,23] have demonstrated that ITCs inhibit the growth of several microorganisms, such as the fungi *Rhizoctonia solani*, *Sclerotinia minor*, *Sclerotinia sclerotiorum*, *Alternaria brassicicola*, and *Fusarium oxysporum*, which can have a detrimental role in agriculture, since they are known to be plant pathogens [20].

ITCs owe their biocidal properties to the strong electrophilicity of the carbon in the isothiocyanate group, which then tends to show high reactivity towards thiols, amines, and alcohols, resulting in the formation of dithiocarbamates, thiourea, or O-thiocarbamate derivatives [20]. Given the presence of sulfhydryl groups in proteins, contact with ITCs can cause mutations in vivo, which in turn can interfere with regular biochemical processes [16,24].

Despite its undoubted advantages, biofumigation also presents limitations. Some impracticalness is related to the fact that it is a time-consuming practice, in which fields need to be firstly cultivated with *Brassicaceae*, to be used as green manure crops, thus determining the non-availability of plant biomass in the off-season. Other cons are represented by the low control of the amount of glucosinolates/myrosinase delivered to the soil [25], and the significant frequency of ineffective outcomes due to the large amount of variables at play [26]. Then, another main issue related to biofumigation is that volatile ITCs are non-persistent, so that the long-term control on the soil treatment is very low. Some improvements have been proposed over time, such as the use of powdered Brassica “flours” or dried pellets [25], to overcome some of these limitations. De Nicola et al. even proposed the use of biofumigating liquid formulations based on *Brassicaceae* vegetal oil extracts from defatted seeds’ meal, which were emulsified in water [27].

However, research on the controlled release (CR) of chemicals and pesticides in agricultural soil has kept attracting a major effort in this context, as being able to modulate the rate of delivery of (bio)active molecules would greatly improve the performances and effectiveness of most treatments.

Several solutions have been proposed to find suitable materials to act as carriers for the CR of different classes of active chemicals [28,29,30,31,32,33,34,35], but probably the main and most effective among the explored alternatives is arguably the use of hydrogels based on biocompatible natural polymers [36,37,38,39]. Several polymers, such as chitosan, modified celluloses, or alginate, to name a few, have been proposed to synthesize gelled matrices that can be conveniently used to encapsulate (bio)active chemicals, provided that they show some hydrophilicity. The high water content of these gels is often an added value to agricultural soil treatment, while the delivery rate can be controlled by tuning the physicochemical release mechanism. Alginate-based hydrogels, in particular, have been investigated with several other purposes, even introducing grafting or other modifications to the polymeric network [40,41].

An innovative alternative to traditional biofumigation, which could improve the control on ITCs delivery and their persistence, could be the direct introduction of ITCs into agricultural soils as loaded into some CR carrier. However, their simple loading into CR hydrogels is impractical due to their poor water solubility. Thus, in this work, ITCs-based microemulsions (based on ionic or nonionic surfactants) were developed, which can be loaded into porous polymer-based hydrogel beads based on sodium alginate (ALG) or sodium carboxymethyl cellulose (CMC).

Microemulsions can be a quite obvious viable solution for the delivery of water-insoluble active chemicals, and, in fact, there exist formulations that were developed and proposed for the release of pesticides against fungi, bacteria, insects, etc.

Song et al. [42] reported on microemulsions based on thiamethoxam and acetamiprid, two insecticides for the treatment of rice cultures. Leng et al. [43], on the other hand, developed microemulsions based on difenoconazole/propiconazole with xylene and methanol that showed antifungal activity against *Rhizoctonia solani*, while Davis et al. [44] published research on microemulsions of methyl and ethylene glycol esters of pelargonic acid to be used as nematocides.

Similarly, microemulsions, nanoemulsions, and also nanoparticles that include ITCs have previously been developed and proposed, even if they were mainly targeted as anti-cancer or antibacterial treatments, together with the many examples of drug delivery systems exploiting surfactant- or lipid-based nanovectors for the release of several active chemicals [45,46,47,48,49]. Within the vast literature published on the topic, Yi et al. [50] reported on PVA films loaded with the essential oil of papaya seeds, which is mainly composed of benzyl ITC. Uppal et al. [51] proposed the use of cerium oxide nanoparticles loaded again with benzyl alcohol, as an antibacterial system, whose effectiveness was assessed against *E. coli* and *S. aureus*. Finally, Kim et al. [52] reported on an emulsion composed by ITC-doped olive oil, nonionic surfactant, and water mixed with sodium alginate to form gelled beads for the oral delivery of allyl ITC in humans.

Hence, to the best of our knowledge, this is the first work that reports on ITCs-based microemulsions, only composed by water, ITC, and surfactant, to be used as fumigants for agricultural soil. Furthermore, loading the selected microemulsion into biocompatible hydrogels grants a feasible application and an increase on the release rate control.

To this aim, three ITCs were selected, namely ethyl isothiocyanate (Et-ITC), phenyl isothiocyanate (Ph-ITC), and allyl isothiocyanate (Al-ITC), which constitute a comprehensive range of possible different chemical structures (aliphatic, aromatic, unsaturated) naturally occurring from the enzymatic hydrolyzation of glucosinolates. Then, three different surfactants were selected to formulate microemulsions, i.e., sodium dodecylsulfate (SDS), Brij 35 (a C_12_E_23_ alcohol ethoxylate), and Tween 80 (a polyoxyethylene (20) sorbitan monooleate): an anionic and two nonionic amphiphiles. The phase behavior of the nine resulting water/surfactant/ITC ternary systems was explored to select the most promising formulation to be studied further. The selected microemulsion was then characterized by means of dynamic light scattering (DLS) to assess micelles’ size, both right after preparation and at the thermodynamic equilibrium.

Then, “unloaded” and microemulsion-loaded hydrogels based on ALG and CMC were characterized with attenuated total reflectance–Fourier transform infrared spectroscopy (ATR-FTIR) and differential scanning calorimetry (DSC) to confirm the presence of micelles in the gels and to study how the microemulsion/gels interaction affects the gel properties, such as the equilibrium water content (EWC) or free water index (FWI).

Finally, by labeling micelles with curcumin, it was possible using UV–Vis spectrophotometry to investigate the loading and release kinetics, which are crucial to evaluate the effectiveness of the proposed solution of ITCs’ CR for the suppression of pathogenic organisms in agricultural soils.

## 2. Materials and Methods

### 2.1. Chemicals

CaCl_2_·2H_2_O (purity ≥ 99%), FeCl_3_·6H_2_O (purity ≥ 99%), Tween 80, Brij 35, sodium dodecylsulfate (SDS, purity > 99.0%), ethyl isothiocyanate (ET-ITC, purity > 97%), Phenyl isothiocyanate (Ph-ITC, purity > 98%), and Allyl isothiocyanate (Al-ITC, purity > 98%) were purchased from Sigma Aldrich/Merck (Darmstadt, Germany) and used without further purification. Food-grade sodium alginate and sodium carboxymethylcellulose polymers were purchased from Sigma Aldrich and Reoper GmbH (Hamburg, Germany). MilliQ Ultrapure (Merck Millipore, Darmstadt, Germany) water was used.

### 2.2. Microemulsions’ Phase Behavior

The phase diagrams of nine different systems were explored to select the optimal formulation, which was characterized in more detail and loaded into the hydrogels for further experimentation. Three surfactants, namely SDS, Brij 35, and Tween 80, were combined with three ITCs, Et-ITC, Al-ITC, and Ph-ITC, to build oil-in-water microemulsions. The critical micellar concentration of the selected surfactants is 8.3 mM for SDS [53], 9 × 10^−5^ M for Brij 35 [54], and 1 × 10^−5^ M for Tween 80 [55]. A relevant portion of each phase diagram was then investigated to assess the monophasic region of each system. All samples were kept at 20 °C in a thermostatic bath and left equilibrating for a month. The selected microemulsion formulation for further experiments was composed (*w*/*w*) by water, 85%; Tween 80, 9.4%; and Et-ITC, 5.6%.

### 2.3. Dynamic Light Scattering

The hydrodynamic diameter of micelles was measured in triplicate on a Malvern Zetasizer Pro Dynamic Light Scattering (DLS) instrument, using a scattering angle of 107°. The autocorrelation functions were fitted by the software using the cumulants method, to calculate the size distribution of samples.

### 2.4. Hydrogels’ Synthesis

Crosslinked hydrogel beads have been prepared starting from 1% *m*/*v* aqueous ALG and CMC solutions. These polymer solutions were added dropwise into saline solutions of, respectively, 0.3 M CaCl_2_ (for alginate) and 0.3 M FeCl_3_ (for CMC) at room temperature. These concentrations were preliminarily optimized to obtain gel beads that were mechanically resistant to handling. The promptly formed hydrogel beads (5–8 mm diameter) were then magnetically stirred for 15 min and then taken out from crosslinking solutions, washed with distilled water (to remove any unreacted metal ions from the gels’ surface), and stored in polyethylene containers, at their equilibrium swelling degree in a slight excess of water [56].

### 2.5. Loading Kinetics

A solution of 0.5 mg/g of curcumin in Et-ITC was prepared. The obtained solution was then used to prepare the microemulsion, with the same formulation reported in Section 2.2. ALG and CMC gels were loaded simply by immersing them in the curcumin-doped microemulsion using a gel/microemulsion 1:1 ratio (*w*/*w*). At regular time intervals, the loading nanofluid was sampled and analyzed using a UV–Vis spectrophotometer set at 425 nm (the wavelength was selected according to the absorbance spectrum of curcumin).

### 2.6. Attenuated Total Reflectance–Fourier Transform Infrared Spectroscopy (ATR-FTIR)

ATR-FTIR measurements were performed with a Nicolet IS50 FTIR spectrophotometer (Thermo Nicolet Corp., Madison, WI, USA), equipped with a single-reflection germanium ATR crystal (Pike 16154, Pike Technologies, Madison, WI, USA) and a deuterated triglycine sulfate (DTGS) detector. The spectra were acquired in the 4000–650 cm^−1^ range with a nominal resolution of 4 cm^−1^, performing 32 scans per sample and using the spectrum of air for background correction. The frequency scale was internally calibrated with a helium–neon reference laser to an accuracy of 0.01 cm^−1^. The OMNIC software (OMNIC software system Version 9.8 Thermo Nicolet) was used for spectra acquisition and manipulation. Hydrogel beads, both “unloaded” and loaded with the microemulsion, were dried and equilibrated until a constant weight before measurement.

### 2.7. Equilibrium Water Content (EWC) Determination

The EWC of the hydrogel beads was measured gravimetrically by completely drying each sample and weighing it before and afterwards. All the samples were left equilibrating beforehand by releasing excess water. EWC content was then calculated through Equation (1) [57]:

(1) $$EWC=\frac{W_{wet}-W_{dry}}{W_{wet}}\times 100$$

where *W*_wet_ is the weight of the swollen hydrogel and *W*_dry_ is the weight of the completely dry hydrogel (i.e., of the sole polymeric network).

### 2.8. Differential Scanning Calorimetry (DSC) and Free Water Index (FWI)

DSC measurements were performed to calculate the free water index (FWI) of gel systems and carried out on a DSC Q1000 (TA Instruments, Leatherhead, UK), using sealed aluminum pans under an inert nitrogen atmosphere (nitrogen flow: 50.0 ± 0.5 cm^3^/min). The samples were equilibrated at −60 °C for 8 min, then heated from −60 °C up to 25 °C at 1 °C/min. The calculation of the FWI from the enthalpy of fusion values (obtained from the integration of the DSC curve peak around 0 °C) was performed according to Equation (2) [58]:

(2) $$FWI=\frac{{\Delta H}_{fus(exp)}}{EWC\cdot {\Delta H}_{fus(theo)}}$$

where ΔH_fus (exp)_ (J/g) is the experimental value of enthalpy variation relative to the melting of frozen free water, and ΔH_fus (theo)_ (333.1 J/g) is the theoretical value of enthalpy of fusion for bulk water.

### 2.9. Release Kinetics in Water

ALG and CMC gels were first loaded with the curcumin-labeled microemulsion, prepared using the same procedure outlined in Section 2.5, by immersing them for 24 h in a gel/microemulsion ratio of 1:5 (*w*/*w*). Subsequently, they were immersed in water, using a gel/water ratio of 1:1 (*w*/*w*). Water in contact with the gel was sampled at regular time intervals and analyzed using a UV–Vis spectrophotometer set at 425 nm (as already carried out to measure the loading kinetics, and described in Section 2.5), to observe the release of curcumin-labeled micelles from the gel.

### 2.10. Scanning Electron Microscopy (SEM)

Scanning electron microscopy micrographs of ALG and CMC pristine gels were taken using a Quanta 400 SEM apparatus (FEI Company, Hillsboro, OR, USA) operating at a voltage of 20 kV. The hydrogel samples were freeze-dried to allow their investigation in high-vacuum conditions. Subsequently, they were placed onto stubs, with the help of a conductive bi-adhesive tape, and they were sputtered with gold to make them conductive as well. Two magnifications were used, i.e., 70× and 600×, respectively, to observe gels’ porosity at different length scales.

## 3. Results and Discussion

### 3.1. Development and Characterization of ITCs-Based Microemulsions

Firstly, the ternary phase diagrams of the nine systems, obtained by combining the three surfactants and the three ITCs selected, were explored to identify monophasic regions and to select the optimal microemulsion to be investigated in more detail.

The study of phase diagrams was limited to the portion relevant to this work, i.e., the water-rich corner, where the surfactant concentration was lower than 20%. With the sole exception of SDS (which was nonetheless included in the study due to its high emulsifying power), the other two nonionic surfactants considered for this work are biodegradable and relatively ecofriendly. Moreover, Tween 80 is a quite common emulsifier in pharmaceutical drug delivery, as a further confirmation of its biocompatibility and low toxicity [59,60,61,62]. However, the aim of the phase investigation was to find and develop a formulation where the ITC concentration was maximized, while keeping the surfactant concentration low enough.

**Figure 1:**
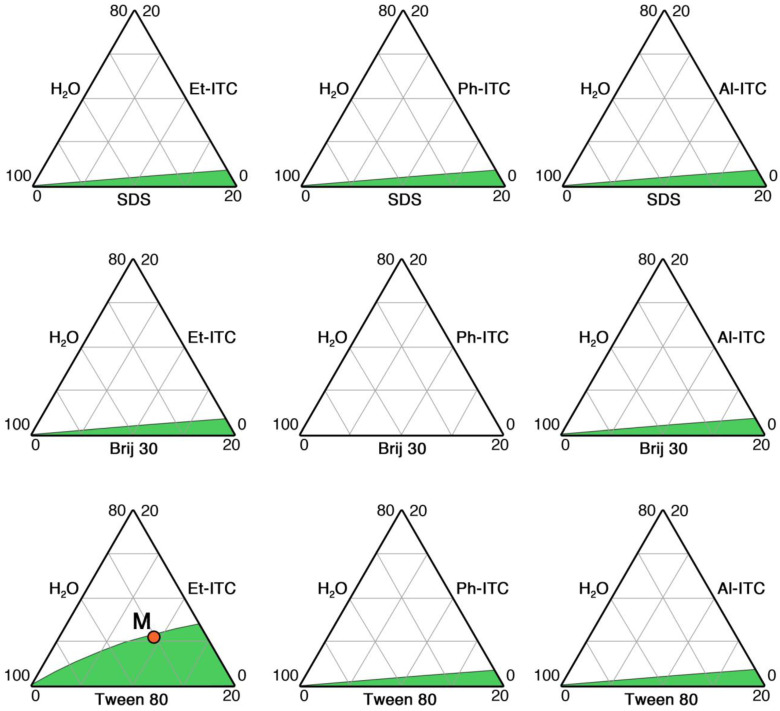
Phase diagrams of the nine selected systems. Only a relevant portion of the whole phase diagram of each system was explored, i.e., the water-rich corner. The green areas indicate the monophasic regions, where oil-in-water micelles are formed. The red *M* point highlights the position of the finally selected microemulsion on the phase diagram.

Figure 1 shows the phase diagrams of the nine investigated systems, which share a very similar behavior—with a few notable exceptions—independently from the surfactants’ and ITCs’ chemical nature. In fact, most of the explored systems are characterized by a limited monophasic region (green areas in Figure 1), where stable microemulsions are spontaneously formed, but where the maximum ITC concentration does not exceed 3%. On the other hand, the water/Brij 35/Ph-ITC system is biphasic for all explored concentrations, meaning that no stable microemulsion could be formed in this case, likely due to the inability of the linear nonionic surfactant to interact with the aromatic ITC, which surely is the one with the most prominent steric hindrance among the three considered. Finally, the phase diagram of the water/Tween 80/Et-ITC system was the most promising in this context, showing a large monophasic area, where stable microemulsions could be formulated, with an ITC content up to almost 8%. This is in agreement with the fact that Et-ITC is the simplest and smallest molecule among the considered ITCs, and Tween 80 is a branched nonionic amphiphile that can be particularly effective in building microemulsions even in the absence of a cosurfactant, which could help in modulating micelles’ interfacial curvature. It is worth noting that in the biofumigation context, aliphatic ITCs were assessed to be more effective than aromatic ones against soil-borne pathogens [17]. Thus, a microemulsion formulation was identified in the water/Tween80/Et-ITC system according to the aforementioned constraints, i.e., arbitrarily keeping the surfactant concentration below 10%. The selected formulation is the one already reported in Section 2.2: water, 85%; Tween 80, 9.4%; and Et-ITC, 5.6%, and it is graphically represented by the *M* point in the bottom-left phase diagram in Figure 1. It is worth noting that the Tween 80 concentration is about 7 × 10^−2^ M, i.e., more than three orders of magnitude above its critical micellar concentration (see Section 2.2).

**Figure 2:**
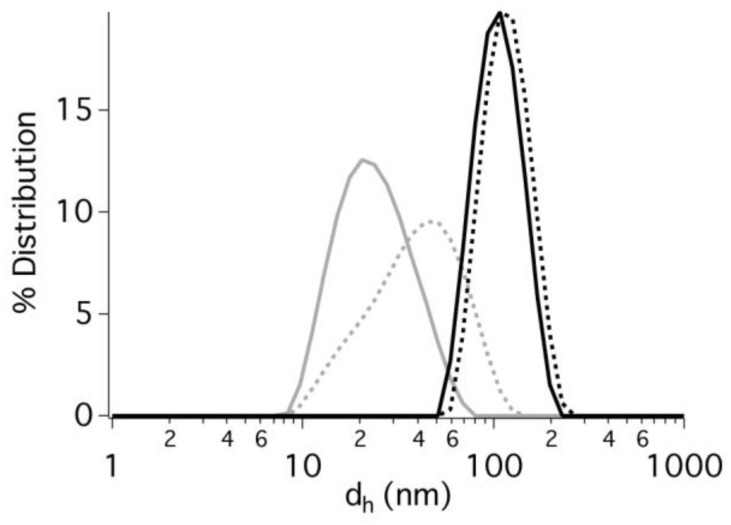
Size distribution measured through DLS analyses. (Solid gray line) Freshly prepared microemulsion; (Solid black line) equilibrated microemulsion; (Dashed gray line) freshly prepared curcumin-doped microemulsion; (Dashed black line) equilibrated curcumin-doped microemulsion.

This system was then characterized by means of DLS analyses to determine micelles’ size and size distribution. Since it was observed that the initially transparent microemulsion progressively tended to look more opaque, until an equilibrium point was reached after approximately two weeks, DLS measurements were performed on both freshly prepared and equilibrated samples. Figure 2 and Table 1 confirmed the expectations, as the average hydrodynamic diameter of the micelles moved from 34 nm for freshly prepared samples to 101 nm at the thermodynamic equilibrium. Also, the polydispersity of the system changed from 0.2, a common value for microemulsions, to 0.01, indicating a much narrower size distribution. This suggests an Ostwald ripening-like evolution of the microemulsive system, where a broad population of averagely smaller micelles changes to a narrower distribution of larger aggregates, likely due to the disruption of smaller micelles that leads to the growth of the bigger ones. Overall, micelles are quite large, so that this system can be considered at the size limit between microemulsions (usually reported as 5–100 nm) and nanoemulsions (>100 nm).

**Figure 3:**
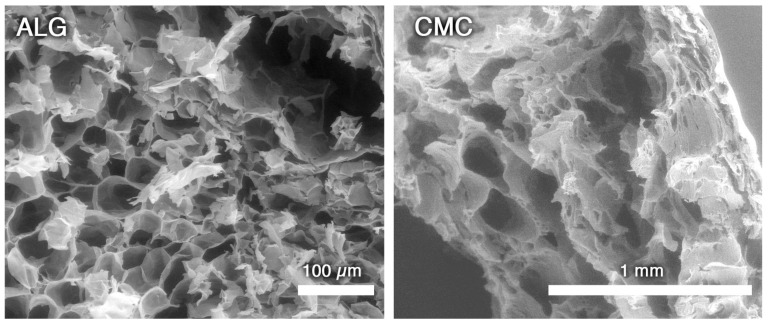
SEM micropictures of ALG and CMC “unloaded” hydrogels. Pores are clearly visible at different magnification in both cases. Gel beads were cut open with a scalpel, in order to expose their inner structure.

However, DLS analyses confirmed that micelles, although large, are small enough to freely diffuse in and out of gels’ porosity, as clearly observable looking at SEM micropictures taken on the inner structures of cut-open pristine ALG and CMC hydrogel beads (Figure 3). In fact, both ALG and CMC gels show sponge-like structures characterized by large interconnected pores of about 20–200 µm.

According to these findings, hydrogels’ loading could be performed simply by immersing a given amount of gel beads in the microemulsion and waiting long enough for micelles to diffuse into gels’ porosity until an equilibrium was reached between the inside and the outside of beads.

### 3.2. Microemulsion-Loaded Hydrogels

A thorough characterization of the pristine hydrogels has already been reported in a previous paper [56], where ALG- and CMC-based beads were also characterized by means of ATR-FTIR spectroscopy and thermogravimetric analyses (TGA). It was found that pristine ALG and CMC hydrogels showed weight loss due to water evaporation up to 120 °C, while the ions’ crosslinking effect could be noticed particularly in the 150–300 °C range, where polymer degradation occurs. In the case of the ALG gel, two degradation peaks in the 160–350 °C range were evidenced, one likely relative to weakly-bound residues and the other (at higher temperatures) relative to the thermal disruption of the highly crosslinked residues.

Differently from other authors that proposed alginate hydrogels combined with microemulsions for other purposes, the polymers were not directly dissolved into the microemulsion [63,64,65]. Even if this could be a way to maximize the loading efficiency, it could actually destabilize the colloidal system, leading to a phase separation during the gelation process. Conversely, as reported in Section 2.5, the gels were loaded simply by direct immersion in the microemulsion. It is worth pointing out that, with the Tween 80 concentration being sufficiently far from the critical micellar concentration, it is also crucial to avoid possible phase separation in this case.

The loading kinetics was measured to assess both the rate of micelles’ diffusion inside the gel matrix and the maximum extent to which the microemulsion could be entrapped into the two different hydrogels, respectively, based on ALG and CMC. To this aim, the concentration of micelles in the microemulsion put into contact with gel beads was quantified over time, in order to measure how this decreased as a result of micelles’ diffusion into the hydrogels. Theoretically, the initial micelles’ concentration in the microemulsion, C_0_, should decrease until the equilibrium is reached when the micelles’ concentration inside the gel equals that outside the gel. The final theoretical concentration, *C*_min_, represents a minimum and can be defined as follows:

(3) $$C_{min}=C_{0}\frac{W_{µE}}{W_{µE}+W_{GEL}}$$

where *W*_µE_ is the weight of the microemulsion in contact with the gel, and *W*_GEL_ is the weight of the water amount inside the whole set of gel beads, equal to EWC × *W*_beads_ (*W*_beads_ is the weight of all the hydrogel beads immersed in the microemulsion; for EWC, see Table 2). Now it is possible defining the loading efficiency, LE%, as follows:

(4) $$LE\%=\frac{\frac{\left(C_{0}-C_{t}\right)}{W_{GEL}}W_{µE}}{C_{min}}\times 100$$

which can be simplified as follows:

(5) $$LE\%=\frac{\left(C_{0}-C_{t}\right)}{C_{0}}\times \frac{W_{µE}+W_{GEL}}{W_{GEL}}\times 100$$

where *C*_t_ is the micellar concentration measured at time *t*. At infinite time, i.e., at the equilibrium, the maximum LE% is obtained.

Measuring micellar concentration is not straightforward; thus, a spectrophotometric method was set up by doping micelles with curcumin, as described in Section 2.5. Contrarily to Tween 80 or Et-ITC, whose absorption in the UV–Vis range is negligible, curcumin has an absorption band centered at about 425 nm [66], which was conveniently used to build a calibration line (not shown here for the sake of conciseness) at 0.5–2 µg/g (curcumin/nanofluid) with R^2^ = 0.9996. The hydrophobicity of curcumin grants that its concentration in water is negligible compared to its concentration in the micelles. Moreover, the solubilization of curcumin into Tween 80/Et-ITC micelles was confirmed by DLS measurements (look at the dashed curves in Figure 2), since, in the presence of the marker, supramolecular aggregates in the freshly prepared microemulsion were slightly larger (average hydrodynamic diameter of 47 nm) with respect to non-doped micelles (34 nm). This size increase can most likely be attributed to the solubilization of curcumin molecules in the hydrophobic micellar core. Interestingly enough, this difference was mitigated by the structural evolution of the microemulsion, upon reaching its thermodynamic equilibrium, as curcumin-doped microemulsion micelles were found to be just slightly larger than non-doped ones. This (unforeseen) feature actually grants that the behavior and diffusion of doped micelles inside the gels is well representative of that of non-doped pristine micelles.

Therefore, quantifying the amount of curcumin, and knowing the curcumin/microemulsion ratio, allowed us to calculate *C*_0_ (and thus *C*_min_) and *C*_t_.

**Figure 4:**
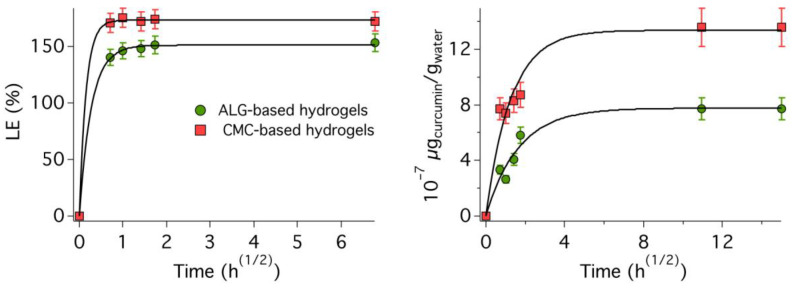
Loading and release kinetics for the two hydrogels. (**Left**) Loading efficiency, LE%, measured over about 48 h; (**Right**) Release efficiency, RE%, measured over about 225 h. In both cases, measurements were stopped when a plateau was reached. Fitting curves calculated using the Weibull model are reported as solid black lines.

As visible in Figure 4, the spectrophotometric data, both for the loading and for the release of the microemulsion, had an asymptotic behavior, showing a plateau after some time. This trend can be conveniently described by a first-order kinetic model [67,68,69], which has the following functional form, if we look at the concentration decreasing in a given volume sample, i.e., the micellar concentration in the microemulsion:

(6) $$Q_{t}=Q_{0}e^{-kt}$$

where Q_t_ is the concentration measured at time t, Q_0_ is the initial concentration, and *a* is a first-order constant, which accounts for the concentration decrease rate. This function can be easily converted into an increase rate, if we focus on the other side of the diffusion path, i.e., the micellar concentration inside the gel:

(7) $$C_{t}=C_{\infty }(1-e^{-kt})$$

where *C*_t_ is, again, the concentration at time *t*, and *C*_∞_ is the concentration at infinite time (at the equilibrium). Equation (7) is obtained from Equation (6) knowing that *C*_t_ = *Q*_0_ − *Q*_t_, and assuming that *C*_∞_ = *Q*_0_.

A lot of different models actually exist that are used to analyze loading and release kinetics involving porous matrices, such as hydrogels or mesoporous particles, including the ones proposed by Higuchi et al. [70], or Peppas and coworkers [71,72,73]. Such models are usually employed as short-time approximations of kinetics curves related to diffusion processes, which can conveniently account for approx. 60% of the whole curve [67]. Peppas model’s equation is expressed as follows:

(8) $$\frac{C_{t}}{C_{\infty }}=kt^{n}$$

where *n* is an adjustable parameter ranging from 0 to 1, with *n* = 1 being the zero-order kinetics model, and *n* = 0.5 retrieving the Higuchi model, which then is as follows:

(9) $$\frac{C_{t}}{C_{\infty }}=k\sqrt{t}$$

The physical meaning of the *n* exponent is related to the kind of diffusion that takes place in the system: for 0.45 < *n* < 0.5, Fickian diffusion is observed; while for 0.5 < *n* < 1, non-Fickian diffusion occurs [74].

Another empiric model that was proposed is based on the Weibull equation [69], where the exponent, *m*, is added to the model, which can be seen as a variation of first-order kinetics:

(10) $$C_{t}=C_{\infty }(1-e^{-kt^{m}})$$

*m* is an empirical parameter, whose linear correlation with the Peppas exponent, *n*, reported in Equation (8) was demonstrated [69].

In the present case, the Weibull function gave the best fitting results, both for the loading and the release kinetics, when *m* = 0.5, thus at the limit between Fickian and non-Fickian diffusion, with a time dependence that resembles the one of the Higuchi model (see Equation (9)). This result is in very good agreement with previous data on micelles’ diffusion with respect to gelled matrices. In fact, several studies reported a mostly Fickian diffusion behavior for cationic or anionic micelles [75,76,77,78], while Tween 80-based micelles usually displayed a mixed behavior more shifted towards non-Fickian diffusion [59,60,61,62]. This might be due to the bulky nature of Tween 80 micelles, and to their irregular fuzzy interface, with respect to much more compact and flat-surfaced SDS or CTAB (alkyltrimethylammonium bromide) micelles, for instance, which somewhat alters their diffusion rate. In Figure 4, kinetics data are plotted as LE% or released amount vs. *t*^(1/2)^, and fitting curves are reported as solid black lines.

Unexpectedly, an LE% higher than 100% was observed for both gels, with LE%_CMC_ > LE%_ALG_, being 173% and 151%, respectively. This means that the final concentration inside both gels is at least 1.5 times higher than the theoretical expected limit. This is, in fact, a positive outcome, since it means that a larger fraction of the active chemical, i.e., Et-ITC, is loaded into the hydrogel. This finding can be attributed to the interaction between Tween 80 and polymer networks inside the gels, which has previously been observed and reported by several authors in previous works [63,64,65]. Poly(ethylene oxide) chains of nonionic surfactants are likely to establish polar interactions with hydrophilic polymers, such as ALG or CMC, thus altering the equilibrium between the inside and the outside of gels, which is thus not only driven by the micellar concentration in bulk water (both outside the gels and inside the accessible pores). It was, in fact, observed that micelles and surfactants also interact with polymeric walls inside hydrogels, just slightly altering their structure and properties, with few to no modifications to the average structure of supramolecular aggregates [79,80].

The loading rate of CMC hydrogel beads was also higher (almost twofold), since *k*_CMC_ = 6.4 ± 1.0 and *k*_ALG_ = 3.6 ± 0.2. Notwithstanding, the loading process was really fast for both systems, as after less than 4 h both gels had reached their plateau (but in about 30 min the whole process was already almost complete). This is in agreement with highly porous gel matrices that act as microsponges, which can be easily and conveniently loaded with ITCs-based microemulsions just by simple immersion.

**Figure 5:**
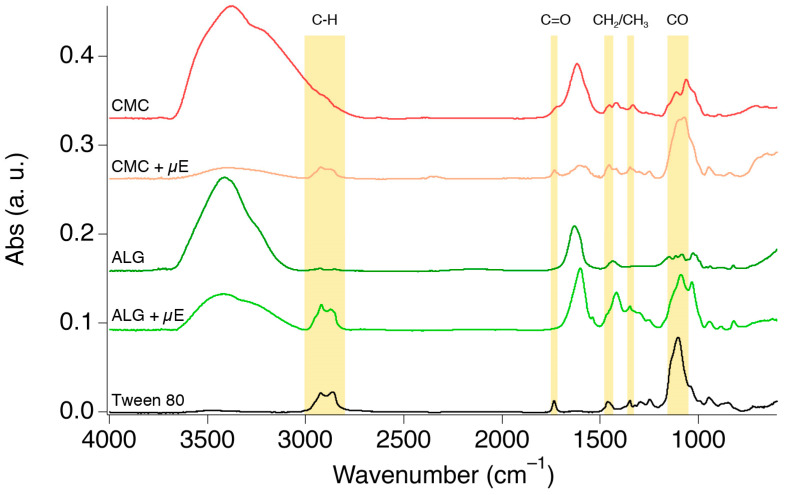
ATR-FTIR spectra of “unloaded” and microemulsion-loaded (“+µE”) CMC and ALG hydrogels, together with a Tween 80 reference spectrum. The hydrogels were dried before the measurements; thus, the presence of the sole surfactant (the only non-volatile compound in the microemulsion) in the loaded hydrogels is further evidence of the migration of micelles inside the gels’ porous matrix. The absorption bands highlighted in yellow are the diagnostic peaks of Tween 80, which can also be spotted in the spectra of the loaded and then dried hydrogels.

ATR-FTIR measurements were then carried out to further assess the presence of micelles inside the gels. Hydrogel beads were dried at room temperature until they reached a constant weight, to remove water (and the volatile Et-ITC), and then analyzed. Infrared spectra of the loaded systems are reported in Figure 5, together with the spectra of pristine “unloaded” gels and the one of Tween 80, as references.

The “unloaded” and loaded gels’ spectra are indeed very similar, yet some significant differences can be noted. The distinctive absorption bands associated with symmetric and asymmetric stretching of C-H at 2928 cm^−1^ and 2860 cm^−1^, esteric C=O stretching at 1734 cm^−1^, bending of aliphatic CH_2_ and CH_3_ at 1462 and 1348 cm^−1^, and C-O-C (ether) stretching at 1100 cm^−1^ which are only visible in the loaded gels’ spectra are, in fact, also present in the Tween 80 spectrum, highlighting the presence of the surfactant in the dried gel matrices. Since the only non-volatile species in the microemulsion is Tween 80, its presence in the hydrogels after the drying process is a further confirmation that the ITC-based microemulsion can be effectively loaded in both ALG and CMC hydrogel beads. Moreover, the high relative intensity of the absorption bands ascribable to Tween 80 in the dried loaded hydrogel spectra is a further confirmation of the high efficiency of the loading process, where the polymer/surfactant interactions seem to boost micellar diffusion into the gels.

Table 2 reports the EWC and FWI values obtained, respectively, as described in Section 2.7 and Section 2.8. Interestingly enough, the EWC is the same for all investigated samples, meaning that neither the chemical nature of the polymeric network, nor the presence of micelles inside the gel beads, significantly affects the equilibrium water content, which is very high (93%, on average), in agreement with the literature for hydrogels based on similar polymers [56,74,81].

The inner structure of both ALG and CMC gels, with mostly interconnected large pores, is also coherent with the high FWI values obtained from DSC analyses, which stay almost constant in the 82–89% range for all investigated systems. And similarly to what observed for the EWC, the FWI also seems to be unaffected by the polymer nature or by the presence of micelles. These findings describe ALG and CMC hydrogel beads as robust and reliable carriers that can be easily loaded with ITC-based microemulsions without altering their physico-chemical properties.

The release kinetics was finally investigated for ALG and CMC hydrogel beads immersed in water. This configuration (release in water) was selected as a model system where it was possible and easy to study and compare the release rate of different gel carriers. Quite obviously, the results in water cannot simply be transferred to the release that would occur when hydrogel beads are embedded in agricultural soils. However, this is a first, crucial, piece of information that allows a comparison between different systems.

Figure 4 (right) shows the concentration of curcumin released plotted vs. *t*^(1/2)^, as already discussed for the LE%. As described in Section 2.9, gel beads were firstly loaded using a gel/microemulsion ratio of 1:5 (*w*/*w*), to maximize the micellar concentration within the gel, and then immersed in water using a gel/water ratio of 1:1 (*w*/*w*), to allow for micelles’ release by outwards diffusion.

Similarly to what discussed above for the loading process, in this case micelles’ diffusion should also theoretically be a concentration-driven process, which would stop when the micellar concentration outside the gel equals that inside the gelled matrix. However, as it was found that the polymer/surfactant interaction actually changes these theoretical equilibria, the absolute curcumin concentration was measured in this case, focusing the analysis on the release rate. As in the case of the loading kinetics, release profiles were also successfully modeled and fitted using the Weibull approach (see Equation (10)), which—as said—is a variation of first-order kinetics. This is, in fact, in perfect agreement with previous works on drugs’ release from alginate gel beads, which was described as a first-order process [82,83,84]. The release efficiency is variable, ranging from 30% to 100%, depending on the given system and especially on the gel’s structure, i.e., the release rate and efficiency is highly decreased for more reticulated and the less porous gels.

Interestingly enough, in the present case, micelles’ release from CMC hydrogel beads is faster and reaches higher values (almost twofold) than release from ALG beads. In particular, *k*_CMC_ is about 26% larger than *k*_ALG_. This behavior surely reflects the fact that CMC hydrogels seem to be also more efficiently loaded, thus eventually containing a higher micellar concentration. However, it also opens up the hypothesis that the porosity in CMC hydrogels is more interconnected and thus easily accessible to micelles’ diffusion into or out of the gel. It has to be said that, overall, the release process is quite slow and the amount of released micelles is not particularly high, especially if compared to loading profiles, but this is most likely due to the same polymer/surfactant interactions that boosted the loading process, which, in this case, slow down the release.

It is worth noting again, though, that release experiments conducted in water (i.e., a controllable model system) are not exactly representative of the actual release that would occur in agricultural soil. In a real application, water in the soil is continuously replenished, following rain or irrigation, in a way that the gel is always in contact with water containing no micelles, promoting constant and, more importantly, controlled release. Furthermore, in soil, polymers’ biodegradation processes would lead to the gradual disintegration of gel beads, resulting in the eventual release of 100% of the active system loaded.

Overall, microemulsion-loaded ALG and CMC hydrogel beads showed very similar physico-chemical properties, in terms of porosity, EWC, FWI, loading efficiency, and release profiles, with CMC-based systems that proved to be slightly more efficient in loading and releasing ITC-containing micelles. Accordingly, both systems seem to present promising features in view of an application in real agricultural soils, with CMC-based hydrogels being moderately preferable. In fact, there exist further considerations that move the choice towards CMC-based hydrogel beads. Some very preliminary microbiological tests showed that CMC/Fe^3+^ beads present some biocidal activity towards some reference microorganisms, namely *E. coli* and *S. aureus*, even in the absence of loaded microemulsion, and this is in agreement with observations by other authors [85,86], who reported on the antibacterial properties of hydrogels and dried powders based on different polysaccharides (CMC, ALG, chitosan, pectin) containing metal ions, among them Fe^3+^. The biocidal properties of iron ions can be explained in view of the metal-catalyzed formation of highly reactive oxygen radicals, as suggested by several authors [85,87,88] or as proposed by Garcìa-Fernàndez et al. [88] who reported on the use of Fe(III) and sunlight to activate Fenton reactions for wastewater treatment. Furthermore, Fe(III) is known to create complexes with thiocyanates and isothiocyanates [89,90,91] (whose antimicrobial activity was assessed [90]), which in the presence of the ITC-based loaded microemulsion could further enhance the biocidal effect of Et-ITC by increasing its persistence in the soil.

An in-depth investigation on these features and a systematic assessment of biocidal properties of these systems both in vitro and in real soils will be the object of further research on this topic.

## 4. Conclusions

As a viable alternative to biofumigation, the possibility of building ITCs-based microemulsions was explored by studying the phase diagrams of nine different systems that combined three surfactants (SDS, Brij 35, and Tween 80), and three ITCs (Et-ITC, Ph-ITC, Al-ITC). The optimal formulation was found to be water, 85%; Tween 80, 9.4%; and Et-ITC, 5.6%, as it proved to be a stable microemulsion characterized by a good ITC/surfactant ratio. This microemulsion was characterized by means of DLS, which showed that micelles evolved from an average size of 34 nm to 100 nm while reaching their thermodynamic equilibrium. Then, the same microemulsion was loaded into presynthesized hydrogel beads made of ALG or CMC crosslinked by means, respectively, of Ca^2+^ or Fe^3+^ ions. The loading efficiency and kinetics were studied via a spectrophotometric analysis, and it was found that both gels can be efficiently and quickly loaded with the microemulsion just by simple immersion of the gels in the nanofluid, in view of the gels’ large and interconnected pores, also evidenced by SEM micrographs, and due to particularly favored interactions between the polysaccharides polymers (ALG and CMC) and PEO chains of Tween 80, further confirmed by ATR-FTIR measurements. In more detail, CMC-based hydrogel beads can be loaded faster and with a higher LE%. It was then shown that loading the ITC-based microemulsion does not significantly affect the structure and properties of hydrogel beads, which maintain the same EWC and FWI. Finally, CMC hydrogel beads also showed a slightly more efficient profile of micelles’ release in water. For this reason and due to the enhanced contributions of Fe(III) to the biocidal properties of the microemulsion-loaded gel beads, the CMC-based system seems to be the more promising for further testing. These will include comprehensive and systematic microbiological experiments, both in vitro and in situ, in agricultural soils, to assess the effectiveness of the developed systems on different pathogens for plants. Nevertheless, this work lays a solid basis for the development of hydrogel-mediated controlled release of microemulsions based on ITCs, which can find their main application in the advanced fumigation of agricultural soils, but also possess the potential to be candidates for use in other fields, from cultural heritage conservation, to food science and nutraceutics, or biomedicine.

## Figures and Tables

**Table 1:** DLS fitting data, showing the average hydrodynamic diameter (d_h_, nm) and polydispersity index, PI, of micelles, right after the preparation of systems or after they were equilibrated for at least two weeks, and in the absence or presence of curcumin (0.5 mg/g with respect to Et-ITC).

Sample	d_h_ (nm)	Polydispersity Index (PI)
Microemulsion (freshly prepared)	34 ± 14	0.2 ± 0.1
Microemulsion (freshly prepared) + curcumin	47 ± 4	0.4 ± 0.1
Microemulsion (equilibrium)	101 ± 1	0.01 ± 0.01
Microemulsion (equilibrium) + curcumin	112 ± 1	0.01 ± 0.01

**Table 2:** Enthalpy of fusion measured through DSC analyses, EWC obtained as described in Section 2.7, and FWI calculated by means of Equation (2) for “unloaded” gels (ALG, CMC) and gels loaded using a gel/microemulsion ratio of 1:1 *w*/*w*. Errors were estimated to be about 10% for the integration of fusion endothermic peaks in DSC thermograms, and about 5% in the determination of the EWC. Errors in the FWI were calculated accordingly.

Sample	ΔH_fus (exp)_	EWC (%)	FWI (%)
ALG	280 ± 14	95 ± 5	89 ± 6
ALG (1:1)	250 ± 13	92 ± 5	82 ± 6
CMC	260 ± 13	94 ± 5	84 ± 6
CMC (1:1)	260 ± 13	92 ± 5	85 ± 6

## Data Availability

The original contributions presented in the study are included in the article, further inquiries can be directed to the corresponding authors.
